# Evidence and Tradition in Dialogue: Biological Sex Variability in Phytomedicine Research as a Foundation for Safety, Efficacy, and Robust Evidence Standards

**DOI:** 10.3390/medicines13020015

**Published:** 2026-04-07

**Authors:** Helen Turner, Chad Jansen, Beverly G. Rice, Tiffany Rivera, Julia Howard, Catherine Brockway, Bianca Parisi, Chaker Adra, Andrea Small-Howard, Alexander J. Stokes

**Affiliations:** 1 Laboratory of Pharmacology and Analytics, Biology and Data Science Programs, School of Natural Sciences and Mathematics, Chaminade University, Honolulu, HI 96816, USA; chad.jansen@chaminade.edu (C.J.); ricebg@hawaii.edu (B.G.R.); tiffany.rivera@chaminade.edu (T.R.); julia.howard@chaminade.edu (J.H.); biparisi@berkeley.edu (B.P.); 2 United Nations CIFAL Honolulu Center, Chaminade University, Honolulu, HI 96816, USA; catherineleebrockway@gmail.com; 3 The Adra Institute, Boston, MA 02215, USA; chaker.adra@gmail.com; 4 AIM-AHEAD Intersex Research Project, Department of Cell and Molecular Biology, John A. Burns School of Medicine, University of Hawai’i at Manoa, Honolulu, HI 96822, USA; astokes@hawaii.edu; 5 Graduate Program, Department of Molecular Biosciences and Bioengineering, University of Hawai’i at Manoa, Honolulu, HI 96822, USA; 6 Graduate Program, Department of Molecular and Cell Biology, University of California at Berkeley, Berkeley, CA 94720, USA; 7 GBS Global Biopharma, Ottawa, ON K2H 7L1, Canada; andrea@gbsciences.com; 8 Laboratory of Experimental Medicine, Department of Cell and Molecular Biology, John A. Burns School of Medicine, University of Hawai’i at Manoa, Honolulu, HI 96822, USA

**Keywords:** SGM, health equity, phytomedicine, sex as a biological variable

## Abstract

**Background:** Incorporating sex as a biological variable (SBV) is recognized as essential for improving the reliability, reproducibility, and generalizability of pharmacological research. This principle is codified in international policies and guidelines, yet implementation remains uneven, especially in phytomedicine. Phytomedicines are a major component of healthcare worldwide, with 65% of the global population relying on them in both regulated and traditional contexts. Globally, phytomedicines are used by males, females, intersex and non-cis gender persons, all of whom may present specific safety and efficacy considerations and warrant full inclusion in pre-clinical to clinical research pipelines. However, in contemporary settings, phytomedicine lags in SBV best practices relative to Western allopathic standards for research design. **Methods:** We conducted a non-systematic review and in silico data mining to quantify sex/gender representation in recent preclinical and clinical phytomedicine studies, complemented by targeted case studies of sexually dimorphic safety/efficacy. We also summarize the historical role of women and gender-diverse people as users and providers within Traditional and Integrative Medical Systems (TIMSs). **Results:** Across rodent and human studies, females are under-represented relative to males, and sex is rarely reported for cell lines. Intentional inclusion of intersex and other gender-diverse populations is largely absent. Case studies illustrate plausible sex-associated differences in pharmacokinetics, pharmacodynamics, and adverse event profiles. TIMSs historically address women’s health needs and include substantial participation by female practitioners; however, contemporary SBV practices remain less standardized than in Western allopathic pipelines. **Conclusions:** SBV integration in phytomedicine is needed to strengthen safety, efficacy, and regulatory-grade evidence. Practical barriers include legacy datasets without sex metadata, limited intersex animal models, and uneven resources across settings. We outline feasible, stepwise practices to improve SBV adoption in a manner compatible with TIMS contexts and recommend expanding current guidelines to better support diverse research environments while maintaining scientific rigor.

## 1. Sex as a Biological Variable (SBV) in Pharmacological Research

### 1.1. SBV: Significance and Importance

Sex and gender are health modifiers [1]. Sex refers to the biological characteristics (chromosomes, hormones, and reproductive anatomy) that are typically categorized as male or female. Some people are intersex, meaning they are born with physical or genetic traits that do not fit typical definitions of male or female bodies. Gender refers to the socially and culturally constructed roles, behaviors, expressions, and identities associated with being a man, woman, both, neither, or somewhere along a spectrum. Gender may or may not align with a person’s sex assigned at birth (see Glossary). The inclusion of sex as a biological variable in biomedical research has a relatively recent history. For much of the 20th century, the default assumption in biomedical research was that findings in male subjects could be generalized to male and female sexes. However, growing physiological evidence and regulatory guidance (e.g., NIH, EMA, CIHR) now emphasize SBV to reduce bias and improve external validity via the systematic inclusion of both male and female subjects in biomedical studies [1,2,3], although (as we review below) this binary lens does not fully represent sex and gender variation. In 1992, the U.S. Food and Drug Administration (FDA) documented that women were markedly under-represented in drug studies, and when they were included, the data was often not properly analyzed to assess for sex differences. The 1993 U.S. Revitalization Act mandated an increase in the number of females included in clinical trials, but they remain under-represented in cell-based, animal, and clinical-translational research. A 2014 review specified that out of 2347 preclinical articles, 22% (516/2347) did not specify the sex of the animal, and of those that did, 80% included males only, 17% included females only, and 3% included both [4]. Cell-based studies are also problematic. In 2014, 15.5% of human cell lines and the majority of primary cells and stem cells were sold without sex identification [5,6]. Although cells have a sex [7], which affects many intracellular mechanisms, cell sex is rarely addressed in papers [8] as a variable of importance [9].

The basis for exclusion of females in clinical studies historically has arisen from the concerns regarding confounding effects of hormonal cycles and pregnancy, and from a harm-reduction stance of considering possible effects on undiagnosed and future pregnancies. At the preclinical level, there has been very little attention paid to the origin sex of the thousands of immortalized cell lines used in biomedical, pharmacological, and toxicological research (see Glossary). Murine and other mammalian models have, like human studies, been biased towards male animals due to the perceived confounding effects of estrus. The relationship between study size, cost, statistical power, and ethics results in a drive to use the fewest number of animals possible. This may also contribute to the conduct of single-sex experiments. Non-binary sex is virtually absent from any preclinical study [10] due to overall neglect of this area, the absence of cell models and the paucity of intersex animal models [11,12], which tend to be deployed only when specifically studying a type of intersex variation that they model (e.g., murine models of androgen insensitivity syndrome) [13,14,15].

Bias towards male data and models in biomedicine is consequential and drives health inequities for women because sex-variant physiological and pathological processes are numerous. Hormone level differences between males and females impact drug safety, efficacy, and metabolism. For example, sex hormones interact with other medications by competing for transport and receptor interaction on target cells, inhibiting enzymes, and altering transcription [16]. In intersex people, both intrinsic hormone variations and hormone treatment (which is also prevalent in transgender individuals) will impact drug safety, efficacy, and metabolism. Differences in oral bioavailability relate to sex differences in major intestinal and hepatic metabolic enzymes (e.g., CYP3A4 and CYP1A2), which are differentially expressed between males and females [17]. Sex-variant pharmacodynamics affect drug efficacy and have been well-documented in areas such as anxiety, neurodegeneration [18], cardiovascular health, pain therapy and perception, blood glucose control, and arrhythmia. Adverse drug reactions (ADRs) have been repeatedly reported to be more prevalent in females [19]. For example, in 2001, the U.S. General Accounting Office stated that most recently withdrawn drugs exhibited more severe and frequent side effects in women than in men [20]. The imperatives for consideration of SBV in TIMSs are identical to those for Western medicine. For example, in a 2012 ad hoc pharmacovigilance study across a network of 38 German Complementary and Alternative Medicine physicians, the majority of adverse therapy reactions (56.5%) were documented in women [21].

### 1.2. Binary Limitations of Standard SBV Approaches

SBV efforts have largely focused on male–female binary definitions of sex and gender. The inclusion of intersex and non-binary populations in biomedical research studies [22] is a pressing issue resulting from the lack of standardization in the measurement of gender identity, limited representation of non-binary populations in study samples, and a lack of understanding about the specific health needs of non-binary individuals. Stigmatization, oppression, and persecution of sex and gender minorities, including in countries that practice TIMSs, may, in turn, constitute access barriers. This creates a need in some settings for the development of practices such as sound SOGI data collection and culturally competent consent. Overall, fitting data to binaries limits the impact of research by overlooking the experiences and needs of individuals who do not fit into these categories. These needs manifest both in Western allopathic and TIMS medical settings, and neither has adequate research inclusivity to address the needs of all persons in the population [23,24].

In addition to inclusion at the clinical/epidemiological study level, there is a significant conceptual and technical challenge in the paucity of cell and animal models that are non-binary. Gender is unique to humans; however, intersex is not, and animal models with unique mutations in sex-linked genes exist but are underutilized [13,15] and do not reflect the diverse spectrum nature of intersex variation. There are no cell lines derived from intersex persons, and the development of in vitro models that reflect health status and common medication regimens in wider gender-expansive populations is a remaining technical and conceptual frontier [24].

### 1.3. SBV: Policy Initiatives in the US and Other Regions

**Figure 1:**
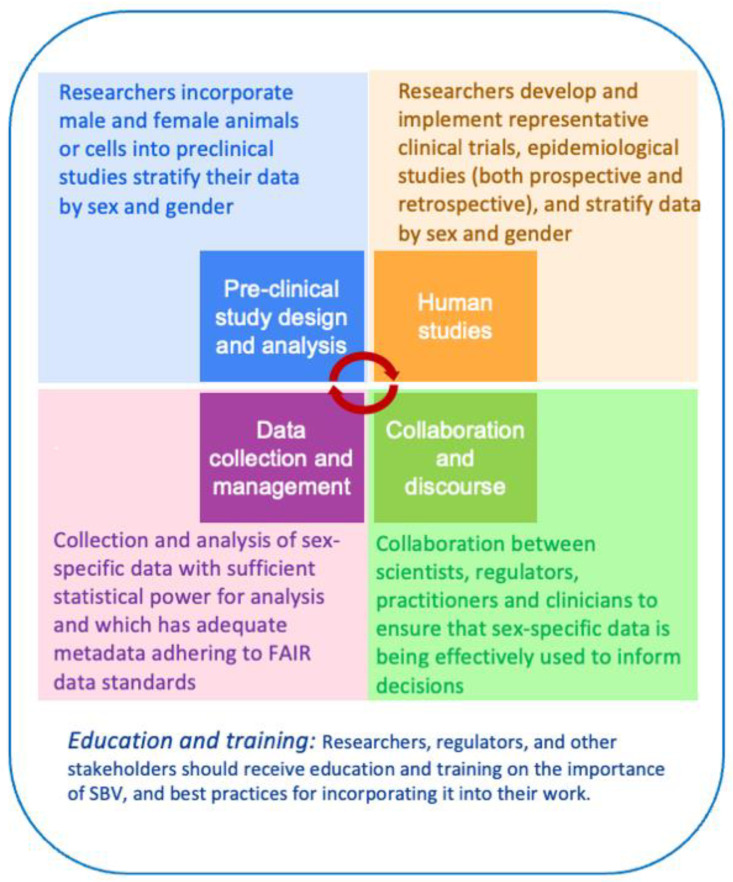
Schematic overview of best practices for consideration of sex as a biological variable (SBV) in biomedical research.

Internationally, efforts have been made to develop and implement policy frameworks that increase the representation of females (but not necessarily non-binary persons) in preclinical/clinical studies and to ensure that data is analyzed by sex. In the 1990s, the U.S. National Institutes of Health (NIH) established policies mandating the inclusion of women in clinical trials, and in 2000, the European Union passed similar legislation. The NIH made it a requirement for grant applications to address the role of sex in the design, analysis, and reporting of preclinical and clinical studies in 2016. However, in early 2025, the White House Executive Orders on DEIA and ‘Gender Ideology’ dramatically rewrote the landscape and eliminated data collection or research on sexes and genders other than male and female [25,26], with likely long-term impacts on the assessment of sex-based variability in biomedicine. Outside the US, the recognition and consideration of sex as a biological variable in biomedical research varies between countries and cultures. The European Commission and Canadian Institutes of Health Research have launched initiatives to promote sex and gender equality in research with accompanying guidelines [27,28]. Japan’s large biomedical research effort has been slower to adopt these practices, but it has recently accelerated the pace of reform and new policy generation. The Chinese government has implemented policies to promote gender equality in scientific research and encourage the consideration of sex differences (e.g., in 2015, the Chinese Academy of Medical Sciences established a “Gender Equality in Medical Science” program to provide training and resources for researchers). There are identified best practices for SBV considerations in Western allopathic drug discovery processes (Figure 1), comprising preclinical study design and analysis, human studies, data collection and management, effective communication and collaboration, and promotion of education and training. Despite these policy frameworks, a persistent sex and gender bias in the under-representation of female or non-binary subjects in biomedical research has remained and may be exacerbated when the therapies in question are Traditional and Integrative Medicine (TIM) phytomedicines. In 2018, more than 85% of member states in the World Health Organization Southeast Asia region reported having national policies in place for the use of traditional medicine; however, none of them referenced the importance of including sex and gender in clinical trials and experimentation [29]. Despite these frameworks, reporting and inclusion remain inconsistent, particularly for phytomedicines, where pipelines and resources differ from conventional drug development.

## 2. Knowledge Gaps Concerning SBV in Phytomedicines Use, Both in Traditional or Western Complementary and Alternative Settings

Traditional and Integrative Medical Systems (TIMSs) are a contemporary healthcare practice globally. According to the World Health Organization (WHO), approximately 80% of the global population relies on TIMSs for primary healthcare needs. TIMS usage reflects the high availability, affordability, and cultural acceptability of these practices in many communities. Phytomedicines have hundreds to thousands of indications across multiple cultures and biogeographies [30,31,32]. Integration of phytomedicines into Western healthcare and the nutraceutical sector is increasing in Western countries such as the US, driven by an increased understanding of efficacy, as well as sociopolitical shifts, the rise of social media, and the spiraling costs of mainstream healthcare. Knowledge gaps include the lack of systematic reviews concerning the adoption, adaptation, or relevance of SBV considerations to phytomedicine research in TIMSs or Western integrative or nutraceutical settings. Moreover, sex-based (or gender-based) differences in the application, usage, provision, efficacy, safety or metabolism of phytomedicines have not been addressed in the literature. In this review, we will focus on SBV considerations in examples of TIMSs where there is a formalized pharmacopeia that links formulation to indication. We will provide an overview of these TIMSs and then evaluate SBV considerations in their research, regulation, applications, usage, and provider populations.

While the evidence base for the action of many TIMSs is based on millennia of observation evidence–iteration cycles, in the contemporary time frame, we are seeing increased frequency of these approaches being placed into Western preclinical-to-clinical pipelines up to, and including, investigation through randomized controlled trials (RCTs). As a component of this narrative review paper, we performed a sampled (non-systematic) literature analysis examining sex representation in cell-based and clinical studies conducted recently in phytomedicine research. We identified four search term combinations for inclusion criteria that we deployed in PubMed (limited to free full-text studies and to studies in English; no date limitations were specified, and the periods covered by the resulting papers ranged from 1970 to 2025):

Group 1: Search Term: *Randomized controlled trial (RCT), plant traditional medicine* (six total in PubMed, all analyzed for sex inclusion, extended to include the NIH Clinical Trial database, resulting in 82 instances).

Group 2: Search Term: *Clinical trial, plant traditional medicine* (746 total in PubMed, 100 most recent analyzed for sex inclusion).

Group 3. Search Term: [*Plant traditional medicine, cell-based study*] or [*plant traditional medicine, cell line*] (895 results, 368 in the last three years (2021–2024), analyzed for sex inclusion).

Group 4: Search Term: [*Murine model studies, plant medicine traditional*] or [*murine mouse model, plant medicine traditional*] (3428 results, 200 most recent analyzed for sex inclusion).

**Figure 2:**
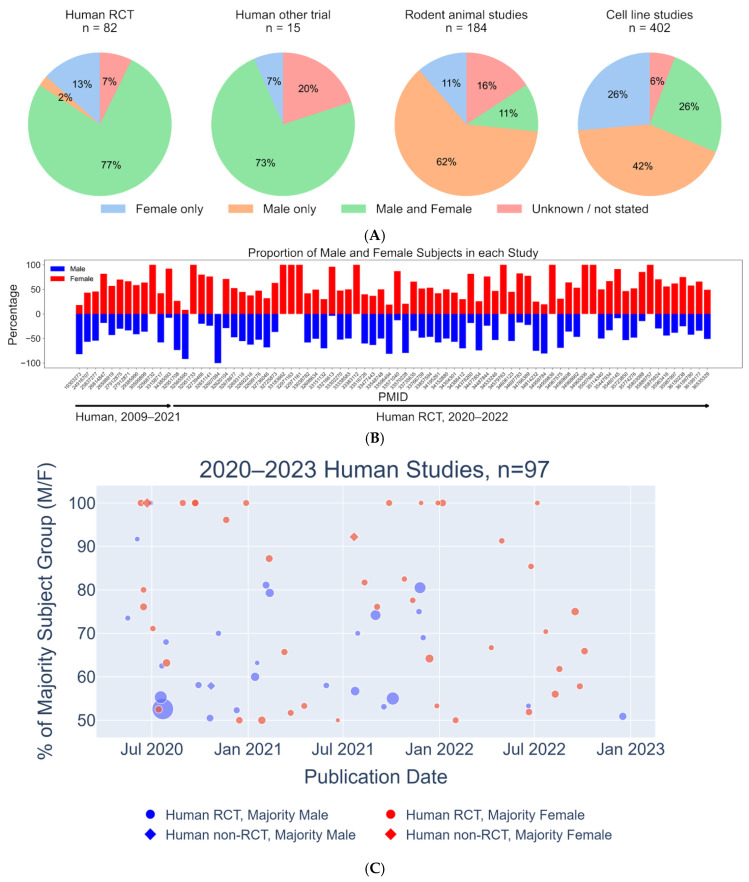

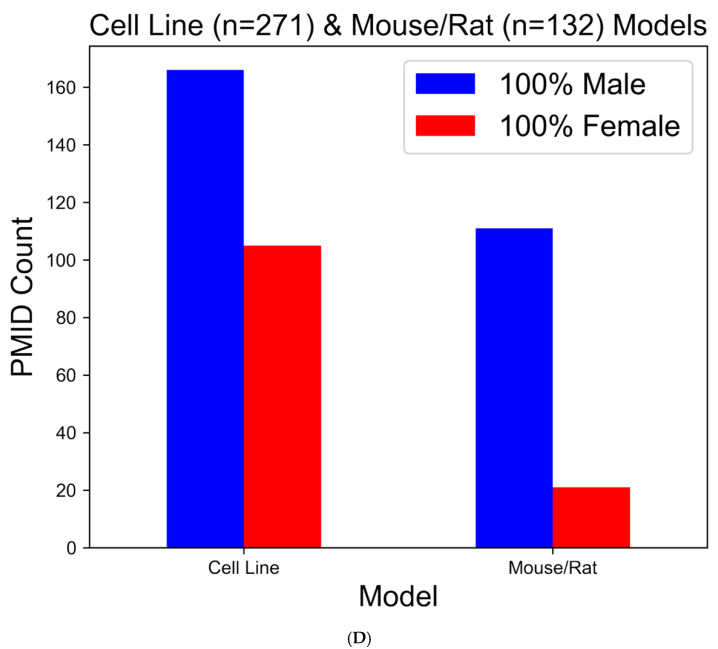
**Sampled literature analysis of SBV considerations in published phytomedicine preclinical and clinical studies.** A sampled (non-systematic) PubMed literature review was conducted to investigate studies involving phytomedicines across various research models, including cell lines, mouse/rat experiments, and human clinical trials. The data was cleaned and standardized to create a structured pandas dataframe. Plots were created using Python packages Numpy, Pandas, Matplotlib, and Seaborn (Version O.13.2). (**A**) **Pie charts illustrating the sex distribution of subjects for each study type represented in the analyses.** Note left panel has 1% unknown. (**B**) **Sex distribution in human RCT studies of phytomedicines (*n* = 97).** This diverging bar chart visualizes the distribution of female vs. male subjects across human non-RCT and human RCT studies included in the sampled literature review (2021–2024). (**C**) **Temporal trends in sex distribution of human non-RCT and human RCT studies included in the sampled literature review (*n* = 97).** This scatter plot provides a temporal exploration of biomedical research articles, with the *x*-axis representing the publication date and the *y*-axis depicting the percentage of the majority group (M vs. F) within each study. Each dot represents a published article, color-coded to distinguish between male and female majority groups. Additionally, the shape of the nodes signifies the experimental model employed, differentiating between human randomized controlled trials and other human-centric models. (**D**) **Sex distribution of cell line and mouse/rat model research.** This grouped bar chart visualizes the distribution of cell line (*n* = 276) and mouse/rat model (*n* = 135) research papers related to medicinal plants and their associated phytochemicals, where participants are either all male or all female, as indicated by the color coding.

The methods for this non-systematic review are as follows: Essential details such as PMID, author, publication year, phytomedicine tested, target disease/disorder, subject type, male/female subject distribution, general exclusion criteria, and summaries of sex-based differences were compiled for each paper. Detailed information was available for 402 cell line studies, 184 mouse/rat experiments, 15 human studies, and 83 human randomized controlled trials (RCTs). Notably, there are relatively few studies, especially in animal and human systems, on phytomedicines compared to the Western preclinical-to-clinical research pipeline. RCTs, other human trials, and rodent studies consistently include approximately 25% (female +include female/total studies) that are either female or inclusive of females (Figure 2A). However, in a study-by-study analysis, the percentage of included females is not always 50% (females/total study participants), even in human trials (Figure 2B,C). At the cell line level, no study explicitly stated the sex of the cell line, but by analyzing them by hand via ATCC and secondary literature searches, we found that male cell lines and male murine models were dominant, with significant female ‘representation’ in cell lines being attributable to HeLa (Figure 2D). In this non-systematic review, we found no evidence of intentional non-binary inclusion in trials and no evidence of phytomedicines ever being analyzed in an intersex rodent model. The non-systematic methodology has weaknesses in consistency, reproducibility, and bias control compared to a systematic review, which would be a desired component of future work to more fully examine these representational issues.

## 3. A Global User Population and Phytomedicines ‘In Motion’ Between Cultures Create Urgency for Consideration of SBV

Phytomedicines are used globally, with estimates suggesting that up to 80% of the world’s population incorporates them into their healthcare [29]. Table 1 presents examples of domestic and diasporic market sizes for an example subgroup of major TIMSs, but not including all TIMSs for reasons of space. This includes people in all regions who use these therapies due to their efficacy, cultural preferences, and long-standing TIMS practices, as frontline approaches in resource-limited settings or as a complement to other forms of healthcare. In numerical terms, this could represent between 4 and 6 billion people globally, inferring that billions of women and millions of non-binary persons are underserved if SBV considerations are not adequately addressed for phytomedicines (see Table 1). Moreover, as phytomedicines are increasingly ‘in motion’ [33] between TIMSs and Western pharmaceutical or nutraceutical markets, SBV-aligned evaluation becomes material to labeling, risk management, and post-marketing pharmacovigilance [34].

## 4. Women and Non-Binary Persons Are Significant User Groups for TIMSs and Phytomedicines Worldwide

As described above, the WHO estimates that approximately 80% of the global population relies on TIMSs for primary healthcare needs, and large populations in developed countries use these medicines. If this translates to billions of users, with 50% or more being women or non-binary persons, then deficits in our understanding of how SBV impacts safety, efficacy, and quality of phytomedicines are highly significant problems. We asked how a lack of understanding of SBV is likely to impact women (and non-binary persons) as user populations for phytomedicines.

Numerous studies place women as more likely to use phytomedical remedies than men across both Western and non-Western settings and both historical and contemporary timeframes. The reasons for this sex difference may include cultural and societal beliefs, limited access to conventional healthcare, and the suitability of traditional remedies for women’s health needs [81,82,83,84]. In some societies, engagement with TIMSs is associated with higher educational attainment and social status (e.g., educated women are overall more likely to take traditional Korean herbal remedies and to visit Korean medicine clinics [85], suggesting that health economic concerns are not exclusive drivers of TIMS usage.

Other global examples include: a 2021 study in the Sámi population of Sweden found that women were more likely to use Sámi traditional medicine and complementary and alternative medicine than men (66.8% to 33.2%) [86]. A 2021 Hong Kong study found that during the COVID-19 outbreak in Hong Kong, women (64.5% versus 35.5% of men) were more likely to use traditional complementary and integrative medicine, including Chinese Herbal medicines as well as acupuncture and dietary supplements to treat COVID-19. In Malaysia, a 2022 study found that women were twice as likely to use Traditional and Complementary medicine (traditional Malay medicine, traditional Chinese medicine and Traditional Indian medicine, in addition to homeopathy) among patients with metabolic syndrome [87].

It appears that there is a strong bias toward women engaging with TIMSs, even when normalizing for the higher level of health-seeking behaviors in most female populations compared to males [88]. Superimposed on these trends, political realities of treatment denial as well as healthcare costs drive women towards phytomedicines for major health concerns. Ralph et al. [89] found that 38.4% of self-managed abortions in a US cohort of >7000 women used inducing herbs. Globally, herbal approaches in poorly managed settings, exacerbated by burgeoning social media misinformation, contribute to the >25 M unsafe abortions estimated worldwide per year [90,91].

Non-binary use of herbalism/phytomedicine is a space where there is an emergent amount of interest and activism. Non-binary and gender queer persons are increasingly finding herbalism and phytomedicine solutions for their specific healthcare issues, including gender-affirming care and general support of wellness and well-being [92]. This shift is likely motivated by the exclusionary nature of hetero-normed healthcare and the often prohibitive expense of mainstream medicine. In addition, U.S. surveys report non-trivial rates of care denials among transgender respondents (e.g., the 2015 U.S. Trans Survey). Such access barriers can increase reliance on over-the-counter or traditional remedies, and robust safety/efficacy data are therefore important for these users [93]. Transgender and gender-expansive populations turn to herbal medicines in a number of scenarios, including as estrogenizing agents [94] and abortifacients [95]. There is a proliferation of websites and community-generated guides for herbalism support of trans/queer well-being, but a PubMed search in 2024 using combinations of keywords for herbalism, herbal medicine, plant medicine, transgender, queer and non-binary revealed <5 relevant published studies, none of which addressed the safety or efficacy of these therapies in these populations. There is a pressing need for a comprehensive data-driven approach to pharmacodynamics, pharmacokinetics, and clinical trial inclusion for transgender and intersex persons, even in conventional Western medicine, and it seems likely that TIMSs will lag that understanding still further. Programs such as the NIH AIM-AHEAD Intersex Analytics initiative and SGMRO (AJS, personal communication) are opening high-visibility conversations on inclusion of intersex and non-binary difference into treatment pathways and outcomes, but a broad understanding of differential pharmacodynamics, side effect profiles, efficacy and safety of mainstream medicines in intersex and non-binary persons is severely limited and non-existent for TIMSs [24].

## 5. Women and Non-Binary Persons Are Significant Provider Populations for TIMSs and Phytomedicines Worldwide

Women have historically played a key role as providers in traditional healing. Numerous studies suggest that this is also the case in the contemporary timeframe [96]. The literature suggests women practitioners outnumber male practitioners, although the ratios vary depending on the type of practice [97,98]. Naturopathy, homeopathy, and Western herbal medicine tend to be practiced more by women, while chiropractic and osteopathy, which require extensive training and a science-oriented curriculum, are more economically and socially accessible to men [97,99,100]. For example, in the UK, 80% of registered Western herbal medicine practitioners were women [100], and 66% of chiropractors were men in 1994 [98]. In India, until the 1950s, Ayurveda medicine was almost exclusively practiced by male physicians, but currently, 80–90% of all students admitted to Ayurveda medical colleges and 50% of people admitted to traditional medical schools are women [101]. In Japan, Kampo is distributed by both traditional practitioners and medical doctors. In the latter, the dearth of women [102] likely translates to a predominantly male prescribing culture, but women practitioners and informal providers seem to often act as mediators to engage the Kampo system for women. In African medicine, women predominate as traditional healers in herbalism, but the two other organized specializations (divination and spiritualism) [103,104,105] are male enclaves.

Different provider pathways and credentialing structures have resulted in distinct gender distributions across modalities. Understanding these distributions may help target SBV training and reporting. There are hegemonic implications of women predominating in TIMS spaces: In some societies, the reservation of healing as a women’s space creates an area of society in which male power is balanced [106]. TIMSs provide an intersectional space that specifically permits the blurring of traditional gender roles [107]. In Western CAM/nutraceutical and herbal medicine provider communities, the strong representation of women likely reflects a similar operating niche not dominated by a medical patriarchy. Of course, this is not an adequate solution overall to the under-representation of women in mainstream Western medicine, but it is a ‘for women-by-women’ sector of informal medical practice that is growing rapidly in the US and in settings with a formalized traditional medicine sector [108].

Representation of LGBTQIA+ individuals in provider populations is critical to mitigating biases and health inequity for non-binary persons. Estimates of non-binary representation in mainstream Western medicine are few, but in the US, a Sexual Orientation and Gender Identity (SOGI) question is included on a matriculation questionnaire for graduating medical students. In 2019, the percentage of graduating medical students identifying as gay or lesbian was 3.8%, and those who had a different gender than that assigned at birth was 0.7% [109]. Intersex status was not addressed. Assuming these are primarily Gen Z individuals, then the overall LGBTQ+ identity in that population is ~20% for comparison [110,111]. There are no published studies on the quantitative representation of non-binary individuals as TIMS providers in the US, but there are examples in some Indigenous communities of the importance of non-binary, gender fluid individuals as traditional healers (at least until the impacts of colonialism, which introduced binary gender norms and suppressed traditional practices and beliefs), which we review briefly here: Māori of Aotearoa/New Zealand have a concept of “whakawahine,” [112] which refers to individuals who embody both male and female qualities and who were recognized as healers in traditional Māori society. In Hawai‘i, māhū [113] healers were/are revered for combining male and female traits in a third gender, akin to the two-spirit individuals in Native American and First Nations communities who often functioned as shaman-healers. Fakaleiti [114] in Tahiti, akava’ine [115] on the Cook Islands and numerous other Pacific third gender groups have been associated with healing. *Mapuche* in Chile [116] are third gender individuals who embody a dualistic medical epistemology in which health is a balance, is about balance; a dualist perspective blending opposing and complementary concepts within the realms of sky and earth, good and evil, and feminine and masculine behavior. In Samoa, there are four recognized cultural genders: female, male, fa’afafine, and fa’afatama [112,114]. Fa’afafine and fa’afatama [117] are fluid gender roles that move between male and female worlds. They play important societal roles in care for elders in the community and sex education, a topic considered taboo in public conversations for male and female genders. Ashtime [118] in Ethiopia are generally healers/storytellers who were assigned male at birth but occupy a third gender space. Zulu sangoma [119] women healers often are in same sex relationships, and the dominant ‘un-African’ [120] narrative about homosexuality is challenged by these women who are respected culture carriers and operate a TIMS sphere in which different sexualities are recognized and embraced [121,122]. Within Europe, Albania, Scandinavia, and Italy have third gender traditions of caregiving. Some cultures have historically excluded or stigmatized gender non-conforming individuals from key community roles, including healing, but in general, the practice of healing seems to be an enclave where non-binary persons are accepted. The ‘otherness’ of third gender persons in some (but not all) Indigenous worldviews likely contributes to the association of these individuals with special power, spiritual power, and human connectedness. Their placement on the boundary of socially constructed gender norms can facilitate otherwise prohibited conversations, which are essential to health and well-being [123].

## 6. TIMS Phytomedicines Are Strongly Representative of Women’s Health Concerns and Evidence of Some Historical Consideration of SBV

As discussed above, women have played a significant role in TIMS practice, serving as community healers and midwives, holding and passing down knowledge, and managing household health [124,125]. While relatively recently, women may have been excluded from the construction of formalized pharmacopeias by patriarchal medical establishments, the centrality of women in the delivery and interactive optimization of TIMSs across long timeframes may well have ensured that two essential conditions for SBV consideration were met: First, the inclusion of women’s concerns in TIMS medical agendas, and second, the inclusion of women as ‘test subjects’ over iterative cycles of observational testing that form the historical evidence base for TIMSs. We review evidence for this inclusivity below. We also note that the prevalence of non-binary and third gender persons in some Indigenous and traditional healing practices is likely to have fostered consideration of non-binary medical needs and built knowledge of efficacy and safety properties of phytomedicines in these populations [126], but published evidence for this is limited.

Several literature reviews prior to this one bear out the strong representation of female-oriented disorder indications within TIMSs. Jiao et al. [127] report ~571 ethnic medicines in contemporary use for menstrual-related disorders. De Boer and Cotingting assessed >200 studies of Southeast Asian phytomedicines for female healthcare and found treatments for fertility, inducing menstruation or abortion, easing pregnancy and parturition, reducing/alleviating menstruation and postpartum hemorrhage, managing parturition and postpartum pain, increasing or inhibiting lactation, and treating mastitis and uterine prolapse [128].

**Figure 3:**
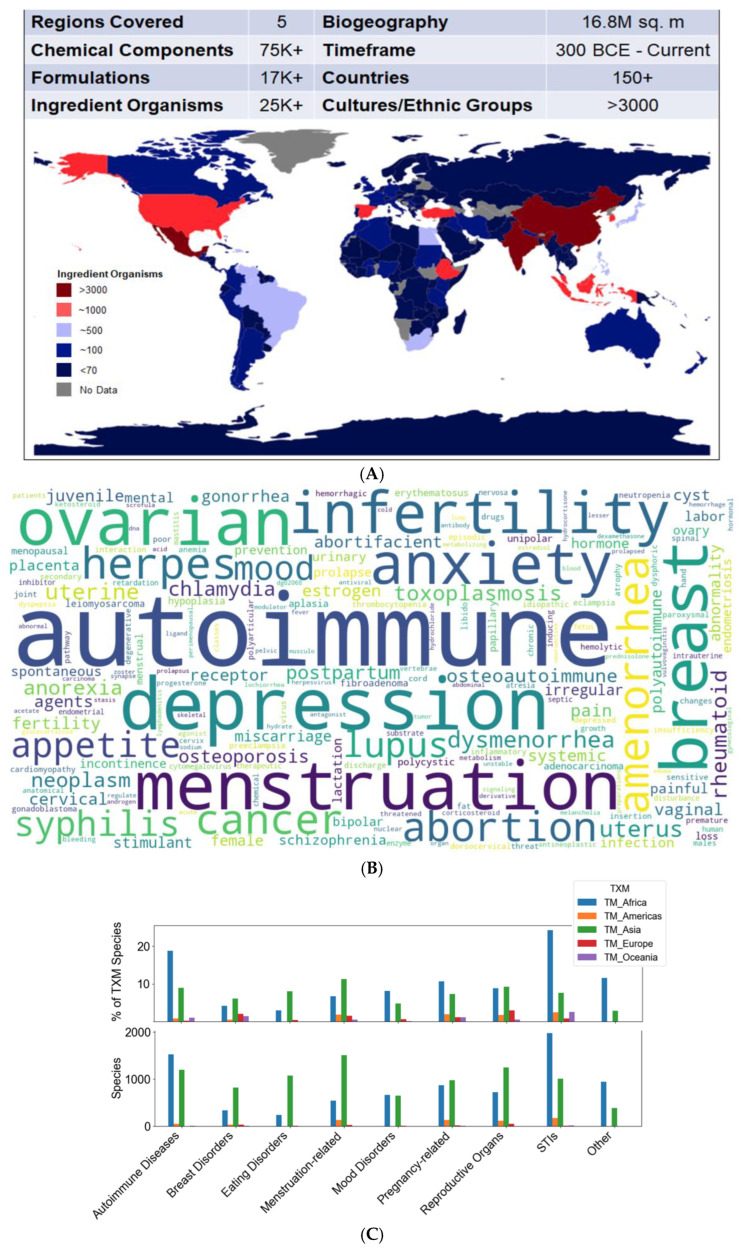

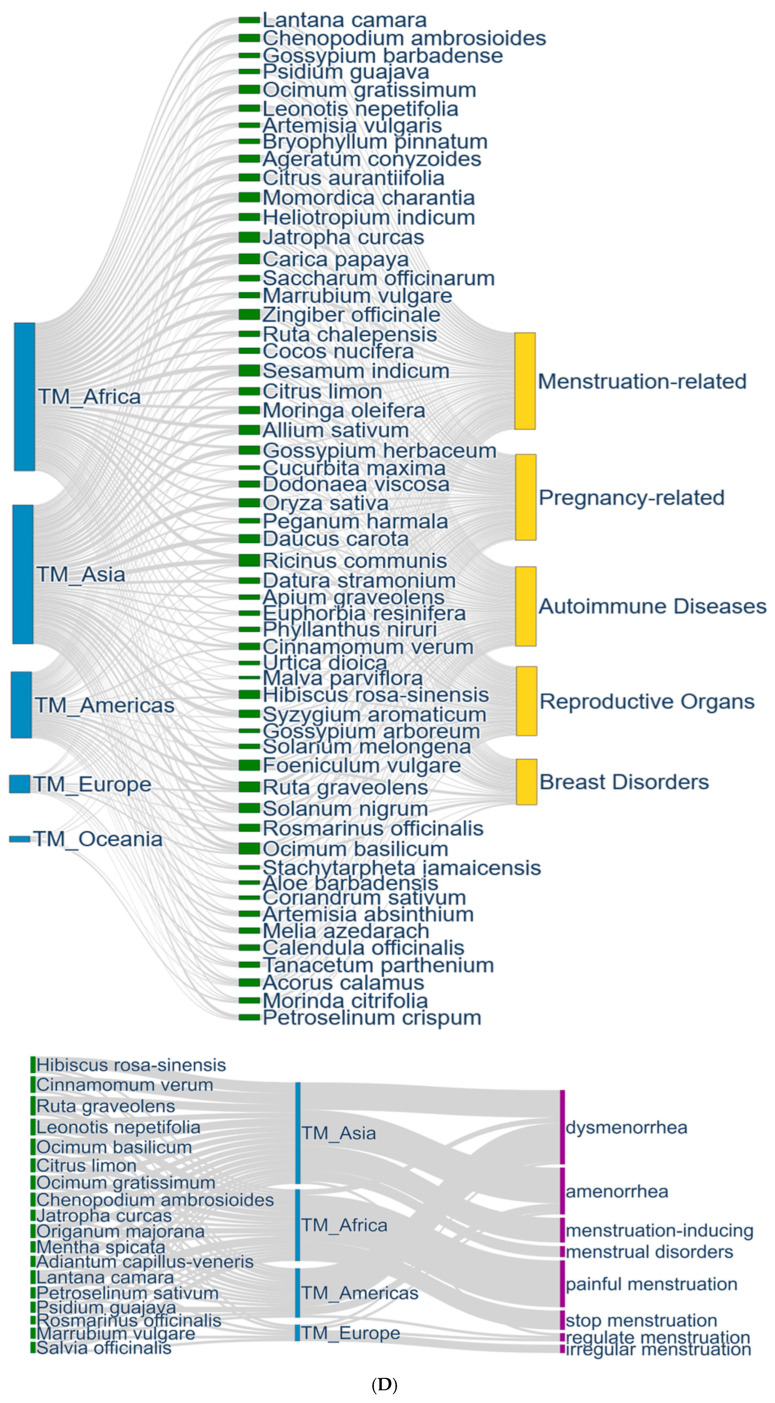

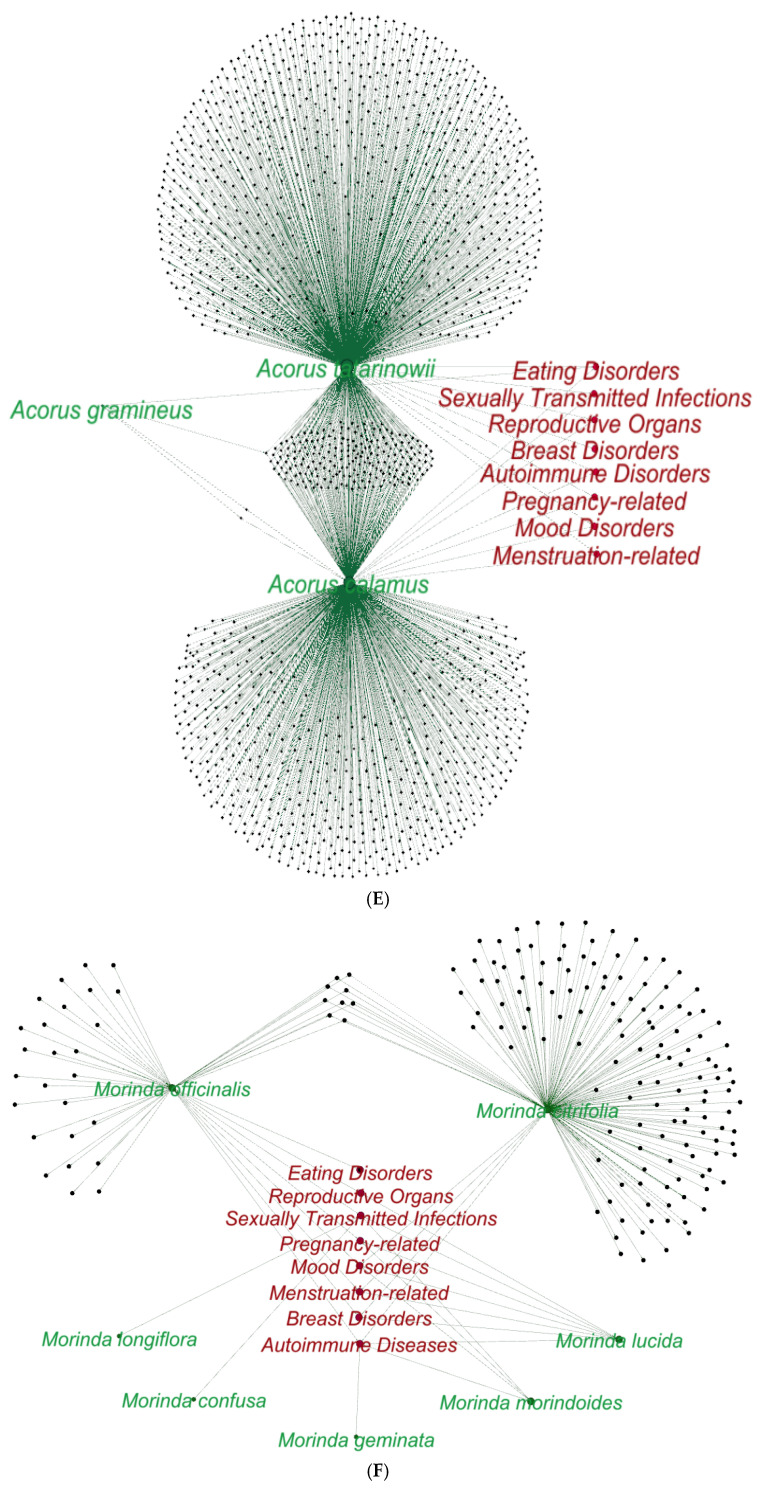
Computational analysis of TIMS representation of female medicine. (**A**) **Overview of in silico integrated meta-pharmacopeia encompassing traditional medicine systems across Asia, Africa, the Americas, Europe, and Oceania.** Of the 195 UN-recognized countries, at least 177 had some phytomedicine association in the meta-pharmacopeia. Meta-pharmacopeia data was interrogated for region, country, formula, chemical compound, and ingredient organisms. Data was analyzed using the Python *Pandas* package. The count of unique-ingredient organisms per country was derived using the ‘groupby’ function of Pandas. The resulting DataFrame of country–species counts was input into the GeoDataFrame function of GeoPandas and plotted to generate the map. The heatmap color codes specific ranges: >3000 (3000–7000), ~1000 (700–3000), ~500 (400–700), and ~100 (75–400), <70 (0–70). (**B**) **Quantitative word cloud of female disorder indications across traditional medicine systems.** The word cloud, generated using Python packages *NumPy, Pandas, Matplotlib, and WordCloud*, shows the frequency of occurrence of various female-related disorders within traditional medicine systems across diverse cultures. The meta-pharmacopeia was interrogated for disorders often associated with women’s health using a dictionary of >50 terms. Word size corresponds to relative frequency. (**C**) **Plant variety used to treat female disorder indications across TIMSs.** The bar plot shows the number of unique plants (ingredient organisms) used to treat female disorder indications captured in the in silico meta-pharmacopeia dataset. The visualization was created using Python packages Numpy, Pandas, and Matplotlib (Version 3.10.8) and provides insights into the variety (species count) and proportion (percentage by region) of plants used in different cultures to treat female disorder indications. The meta-pharmacopeia was interrogated for region, country, formula, species, and indication details. Data was organized into a structured dataframe using the Python package *pandas*. Indications were categorized into distinct groups based on relevant keywords, covering a wide spectrum of women’s health issues, including pregnancy-related concerns, breast disorders, menstruation-related issues, sexually transmitted infections (STIs), female reproductive disorders, immune-related conditions, mood disorders, eating disorders, and other miscellaneous health concerns. Leveraging the ‘groupby’ function in the *pandas* library, the number of species associated with each categorized group was quantified. The two grouped bar plots show the raw count of species for each categorized group across regions, and the percentage of total species within each group relative to the overall unique-ingredient organisms by region. (**D**) **Plants with multi-regional use in the treatment of female disorder indications.** Upper panel. Sankey plot (Python packages Numpy (Version 2.0), Pandas (Version 2.2), and Plotly (Version 5.2)) mapping connections between regions, medicinal plants, and associated women’s health issues. The leftmost nodes represent the 5 regions covered in the meta-pharmacopeia (Asia, Africa, the Americas, Europe, and Oceania), while the middle nodes signify the diverse plant species used to treat at least 3 varying women’s health issues in three or more regions. Thicker lines between the region nodes and plant nodes indicate greater diversity in the type of women’s health issues addressed with the associated plant in the respective region. *Lower panel*. Filtered results including only plants used in >three regions for >3 women’s health issues. (**E,F**) **Network diagrams of specific plant genera associated with women’s health indications.** The meta-pharmacopeia was interrogated for species, associated categorized indications, and associated chemical constituents. The data was organized into a *pandas* dataframe and then input into *Gephi* for visualization. The *Yifan Hu Layout* was employed to plot the network (optimal distance of 100, relative strength 0.2, initial step size o20, step ratio o0.95, adaptive cooling enabled, convergence threshold 1.0E-4, quadtree max level o 10, and theta value 1.2). The network was color-coded by plant (green), categorized indication (magenta), and chemical constituent (black). (**E**) ***Acorus* genus, associated chemical compounds, and indications.** The meta-pharmacopeia was interrogated for all known compound names and PubChem compound identifiers found in the *Acorus* genus and visualized using the software Gephi (Version 0.9.7). *Acorus* calamus appeared to be the only species within the meta-pharmacopeia to be used in all 5 regions for at least 1 of the select women’s health issues discussed here. It has 735 known chemical constituents, while the two additional species in the genus within the meta-pharmacopeia, *Acorus tatarinowii* and *Acorus gramineus*, have 939 and 3, respectively. Between the 3 species, *Acorus calamus* shares 203 known chemical constituents with *Acorus tatarinowii* and only 3 known constituents with *Acorus gramineus.* It is known to have psychoactive chemicals and has also been used as an antispasmodic and carminative. (**F**) ***Morinda* genus, associated chemical compounds, and indications.** This network diagram illustrates the shared chemical constituents and associated women’s health issues of specific species in the Morinda genus, as reflected by our meta-pharmacopeia. *Morinda citrifolia* (also known as Noni in Oceania) has been studied in association with improving joint pain, mobility, physical endurance, immune activity, weight management, osteoporosis, and hypertension.

We used a data analytics approach to examine the representation of female health priorities in TIMS phytomedicine usage. We developed a data platform comprising aggregated phytomedicine formulation, indication and ingredient organisms and targets across multiple TIM systems. This integrated meta-pharmacopoeia developed by our research team is named PhaROS (Phytomedical analytics Research Optimization at Scale), and it provides the capacity to analyze pharmacopeias from Traditional Chinese, Japanese, Korean, Indian, African, and South American medical systems from indication to formulation to chemical composition and targets [129,130]. We use the term ‘meta-pharmacopeia’ as PhAROS unifies multiple pharmacopoeias into a single, higher-order knowledge space, enabling computational reasoning across traditions and regulatory domains. The term ‘meta’ is used here in the same sense as in modern integrative computational sciences, denoting synthesis or abstraction across parallel datasets or frameworks (e.g., metagenomics, metabolomics, meta-analysis). In this computational space, we assembled a data platform summarized in Figure 3A, with included TIMSs covering ~17 M sq. m of biogeography and 178 countries/territories.

To assess the inclusion of female disorders in PhAROS, we first compiled a quantitative word cloud analysis. This depicts the relative frequency of occurrence of various female-related indications in traditional medicine systems across multiple cultures (Figure 3B). In Figure 3C, we show the numerical variety of plant organisms used to treat women’s health issues by TIMSs. The upper panel shows the % of plant species in that medical system pharmacopeia that are included in formulations for the indicated disorders and the lower panel shows absolute numbers of plant species. Figure 3D captures the striking level of agreement between geographically and culturally separated TIMSs on the species of plants with application to women’s disorders. The PhaROS platform also allows us to examine agreement between TIMSs on chemical constituents that are potential mechanistic candidates for efficacy (Figure 3E,F). For example, the Oceanic medicinal plant Noni (*Morinda* spp.) and *Acorus* spp. (best known in Ayurvedic and S.E. Asian medicine) both occur repeatedly in formulations directed to women’s health issues, with a subset of their chemical constituents (shown as black nodes) being shared by multiple species within each group and with documented analgesic, anti-inflammatory, muscle relaxant, and anti-biotic properties.

This type of analysis can streamline the types of preclinical-to-clinical evaluation needed for many phytomedicines by identifying a subset of candidate compounds of interest and by decreasing the resources needed in terms of time and money, which are often drivers of inadequate study size or diversity.

We examined the question of inclusion of women as ‘test subjects’ over iterative cycles of observational testing that are foundational to the non-Western evidence base for phytomedicine efficacy and safety. Within the reproductive medicine space, there has clearly been historical and contemporary consideration of the safety of herbal medicines in pregnancy. Yashoda Devi, a pioneering female Ayurveda practitioner, worked to integrate women’s health issues into Ayurveda practices using observational evidence [101,131]. Historically, certain ‘banned’ herbs during pregnancy have been identified in almost all TIMSs, and the dangers of these medicines have been reaffirmed or extended by modern studies in some TIMSs. For example., Tang et al. evaluated embryo–fetal development and pre- and postnatal growth in a murine study of 20 TCMs prescribed for pregnancy. Maternal effects on side effects, weight loss, litter reduction, implantation failure and fetal resorption and perinatal effects on growth restriction, developmental delay, congenital malformations, and postnatal mortality were observed for some of these TCMs.

There is also a significant risk associated with the gaps between traditional practice and reports that are published in the Western literature. Largehead Atractylodes Rhizome (LAR) is a widely used Chinese medicine in at-risk pregnancies with the intent of preventing miscarriages. Li et al. found decreases in fetal growth and increased likelihood of miscarriage in animal models treated with LAR [132], likely related to suppression of limb development genes [133]. LAR is also prescribed for obesity, stomach aches, and indigestion (based on studies solely performed in male animals) [134,135], which creates a risk for a pathway to female use of LAR through internal medicine prescriptions that may not incorporate evidence about reproductive risk. A 2021 comprehensive review compiled evidence for LAR efficacy in gastrointestinal, immune, antibacterial and cancer indications, but the toxicology section made no mention of reproductive issues [136].

The paucity of evidence around safety is a significant issue for phytomedicines. In a 2012 multinational study (Europe, North America, and Australia) [137], 29.3% of women (*n*  =  2673) reported the use of 126 herbal medicines in pregnancy; 27/126 of these medicines were classified as contraindicated in pregnancy and were used by 20% of women. Only 28/126 were classified as safe in pregnancy, and the remainder could not be classified as safe/unsafe due to a paucity of preclinical or clinical evidence [138]. We suggest that this creates a need for a systematic evaluation of extant studies for sex-based differences in phytomedicine efficacy or safety, as well as prospective approaches to SBV going forward. We searched the literature (PubMed) to find examples of phytomedicine papers where findings illustrate clear sex differences. Preliminary progress on this literature search (Table 2) provided us with a limited number of examples where clear sex-based differences were evident, suggesting that a more exhaustive effort would be of value to the field.

## 7. Summary and Perspectives

Sex and gender equity in the testing and use of phytomedicines is crucial to ensure that treatments are safe and effective for all individuals and has been advocated for in other emerging medical areas, such as nanotherapeutics and biomaterials [155,156]. However, we submit that current inclusivity frameworks that limit SBV to male–female binaries are not adequate or fit for purpose. In the phytomedicine field, SBV best practices (Figure 1) and the accompanying standards for publication, grant funding, and regulatory approval are feasible for well-resourced research communities embarking on new efficacy or safety studies. Standards such as SAGER (Sex and Gender Equity in Research) [157] lay out clear guidelines (Table 3). However, there are limitations and feasibility considerations, specific to the phytomedicine field, that need to be considered in frameworks such as SAGER, which require specific methodological development or other adaptations. The challenge lies in preserving the integrity and intention behind traditional practices and situational awareness of TIMS settings while striving to meet international standards for gender equity in research.

Sex and gender equity in the testing and use of phytomedicines is crucial to ensure that treatments are safe and effective for all individuals. Key priorities for achieving sex and gender equity in this area include:

○ Improving the representation of women and non-binary individuals in clinical trials, animal studies and preclinical cell line studies and development of novel tools, such as non-binary synthetic patient groups, intersex animals and cell lines;
○ Viewing and interpreting data through a lens informed by sex-dependent differences in physiology, pathophysiology, interaction, side effects and the pharmacokinetics and pharmacodynamics of phytomedicines;
○ Achieving sex and gender balance in provider populations, and achieving full representation of female, intersex, and non-binary health concerns in the setting of research agendas and resource allocation for phytomedicine evaluation;
○ Fully considering cultural, political, and economic contexts for phytomedicine research when developing SBV guidelines, and developing resource pools of expertise and financing that enable full participation for researchers outside Western institutions.

**Figure 4:**
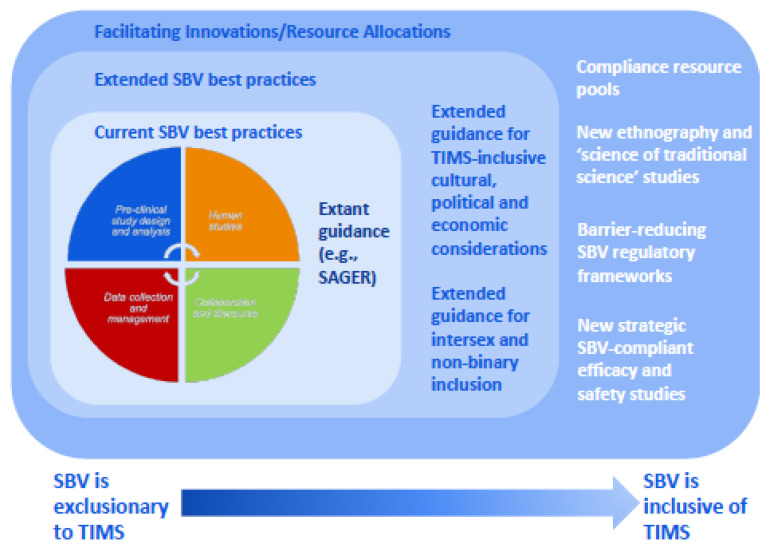
**Expansion of SBV best practices and resource allocation in support of TIMS inclusivity.** This figure presents suggested evolution of SBV best practices in support of TIMS research and practice. The central pie chart details four key areas of current SBV best practices: preclinical study design and analysis (orange segment), human studies (red segment), collaboration and discourse (green segment), and data collection and management (blue segment). Surrounding this, two layers show the needed progression from current practices to extended best practices, and further into facilitating innovations through resource allocation. A directional shift is proposed that fosters and recognizes TIMS sex and gender inclusivity and adapts Western-centric SBV frameworks to better enfranchise TIMS research and practice into SBV. The external rectangles enumerate specific extended regulatory guidelines, research innovations and resource allocations that would start to address cultural, political, economic, and non-binary considerations in SBV both in Western allopathic and TIMS spaces.

Each of these requires, in turn, significant investment of time, money, and scientific expertise. Addressing female under-representation in cell lines, animal models, and clinical/epidemiological studies is a challenging and yet unfinished process. Consideration of intersex and non-binary biology (especially the gender spectrum) opens an additional new frontier for which tools, methods, regulatory frameworks, and cooperation have yet to be developed [24,158]. These areas are as technically daunting, under-researched, and under-resourced [159] for conventional Western drug discovery as they are for phytomedicine research. Figure 4 provides an overview of the multi-faceted challenges and recommendations associated with the achievement of sex and gender equity in phytomedicine research.

Traditional knowledge systems have suffered from epistemicide, where both knowledge and its holders have been systematically erased, and yet a hierarchy of knowledge that minimizes traditional medicine and heralds approaches such as RCT is still widely accepted. We recognize that calls to validate phytomedicines solely through Western scientific frameworks risk reinforcing epistemic hierarchies, but the value of Western frameworks for validation can support TIMSs. A reciprocal approach would instead advocate for mutual integration: studying TIMSs through Western methods, but incorporating TIMS principles such as iterative practice, inclusivity, equity, and democratized knowledge into mainstream medical research.

## 8. Conclusions

We support the international consensus that SBV should be considered at each stage of the drug discovery pipeline, from target identification to clinical trials, and that this responsibility extends from Western drug discovery pipelines to TIMSs. In conclusion, advancing the field of phytomedicine requires a multifaceted approach that acknowledges the full spectrum of biological sexes and gender identities and integrates SBV considerations and methodologies into both culturally centered and Western epistemologies of research into efficacy and safety.

## Figures and Tables

**Table 1:** Global user estimates for phytomedicines.

TIMS	Status	Domestic User Estimate [35]	Diasporic/Transcultural User Estimates Examples	References
Traditional Chinese Medicine (TCM)	TCM was included in Chinese national insurance plans in 1995, and 40% of all medical care delivered in China is based on TCM.	>200 M patients/year.	>3 M Americans/year	[36]
Traditional Korean Medicine (TKM)	Korea has a dual healthcare system that assigns separate licenses, education, and medical facilities to physicians of traditional Korean medicine and Western medicine), with 11 Traditional Medicine Colleges nationally. TKM is often provided to people in rural areas and with low socioeconomic status; however, people with higher educational and socioeconomic status also express traditional remedy preferences. Korean medicine was included in national insurance in 1987.	69.3% of the Korean population (2013 estimate).	High numbers of Korean diaspora in the US, Europe and Asia.	[37,38,39,40]
Japanese Kampo	Kampo was officially recognized by the Japanese Healthcare system in 1961. In 2001, Kampo was incorporated into the core curriculum of all medical schools, and 80% of medical doctors in Japan now prescribe Kampo routinely.	50% of the Japanese population.	User bases in Europe and North America; no robust numerical estimates.	[41,42,43,44]
Traditional African Medicine	Traditional African medicine integrates physical, spiritual, social, and environmental aspects of health. It includes the use of over 5000 species of plants for herbal medicine. The use of traditional African medicine is prevalent due to its efficacy, cultural beliefs, accessibility, and affordability, as well as the perceived limitations of Western medicine in certain communities or regions.	The WHO estimates that ~80% of the African population uses traditional medicine for primary healthcare and has advocated for the inclusion of traditional practices into the healthcare systems of African member states.	Diasporic use of African medicine is prevalent in many countries, for example, dating back to the slavery era in the US.	[45,46,47,48]
Traditional Medicine in Europe	Despite the dominance of Westernized healthcare systems in Europe, traditional medicines, including phytotherapy, retain cultural and contemporary significance. Herbal medicines are often prescribed alongside or as an alternative to conventional drugs with rigorous regulatory standards, e.g., the Traditional Herbal Medicinal Products Directive [49] similar to those for pharmaceuticals, and many are covered by health insurance.	Usage estimates suggest a growing user base for herbal medicines, and a 2012 estimate [50] suggested 100 million (~20%) EU citizens used homeopathic or herbal medicines.	Diasporic use of European herbal medicine is prevalent in many countries where European immigrants have settled.	[49,50,51]
Traditional South American Medicine	South America houses at least 30% of the world’s biodiversity and has an extensive pharmacopeia of phytomedicine used in traditional healing practices and globally.	Includes 40–50 M indigenous persons.	South American medicine is widely used in the US diaspora, and there is a growing interest in its psychedelics as both mainstream and complementary medicines in the US, Europe, and Asia. According to National Survey on Drug Use and Health (NSDUH) data from 2021, approximately 1.2 million U.S. adults reported using psychedelics like psilocybin, LSD, or MDMA in the past year. Solely medical user estimates are not available, but the market for medical psychedelics in the US was estimated at $2.3 B in 2023.	[52,53,54,55,56,57,58,59,60]
Traditional Central American Medicine	Traditional medical systems in Central America (e.g., Maya (Guatemala, Belize), Nahua/Pipil (El Salvador), Lenca (Honduras/El Salvador), Garífuna (Belize/Honduras), Miskito (Nicaragua), Bribri and Cabécar (Costa Rica), Ngäbe and Kuna (Panama)) are widely practiced but are unevenly formalized within national health systems. Several governments have created intercultural health policies recognizing Indigenous healers. No Central American country has a CPT-like national billing system for healers, and public insurance programs typically reimburse biomedical services only.	Estimates range from 50–75% of the 53 M population of Central America [61].	Diasporic usage in the US documented in a number of individual studies [62]. Trends may parallel those of other immigrant populations (see below).	[61,62]
Traditional North American Medicine	Traditional North American medicine includes diverse Indigenous healing systems (Native American, First Nation, Alaskan Native) and Mexican medicine. There is gradual but uneven movement toward formalization and integrated care with Western biomedicine. Within tribal and Indian Health Service (IHS) settings, many clinics collaborate with healers through referral, designated cultural spaces, and inclusion of ceremony within behavioral health programs. Some states have formal mechanisms for reimbursement: Washington and Alaska, for example, allow Medicaid coverage for traditional healing through state plan amendments or tribal agreements. Outside these systems, however, traditional practices generally lack standardized credentialing pathways, CPT billing codes, or explicit insurance recognition, limiting broader reimbursement and integration into mainstream care. As a result, incorporation into Western clinical settings is not yet standardized at the national level. Mexico’s General Health Law recognizes TM as national health heritage and supports intercultural health units, Indigenous midwife programs, and documentation of medicinal plants. Several public hospitals maintain intercultural clinics. Mexico has partial but not standardized mechanisms for inclusion of traditional medicine within public insurance frameworks.	Includes ~7.5 M indigenous persons. Canadian statistics (2024) suggest 86% of First Nations people living off-reserve, 82% of Inuit, and 70% of Métis placed importance on having health-care services that support Indigenous traditional medicines, healing and wellness practices [63]. A total of 130 M potential Mexican users; usage estimates are sparse but suggest >2.5% [64].	One review points to highest usage on or near reservations for North American Indian Tribal medicine [65]. Urban Indian organizations report high demand [66,67]. Latinx data support strong diasporic use of Mexican medicine (e.g., 300 first-generation Mexican immigrants in southern Arizona found that 92.3% reported continued use of at least one domain of Mexican traditional medicine; wider statistics suggest >31% of the general Mexican population in the US engages with TM) [68].	[63,64,65,66,67]
Traditional Indian Medicine	A government ministry was created in 2014 to promote Ayurveda, Yoga, Naturopathy, Unani, Siddha, and Homeopathy. These systems are officially recognized and integrated into India’s healthcare system, with over 800,000 licensed practitioners. Traditional medicine is widely used in both rural and urban areas and is covered under India’s national health policies.	~77% of Indian households use AYUSH systems (2014–2015 estimate); ~300 M users estimated in India.	Ayurveda is used widely among the Indian diaspora globally. For example, a 2007 NCCIH survey found ~240,000 users in the US. The global Ayurvedic market is growing and estimated to exceed $14 B by 2028, including users in the US, Europe, Africa, and Southeast Asia.	[69,70,71,72]
Oceanic Medicine	Traditional Oceanic medicine (Indigenous healing systems across Polynesia, Micronesia, Melanesia, and Hawai’i, Aotearoa, Australia, etc.) is widely used but only partially formalized within national health and insurance systems. The WHO encourages Pacific Island countries to document and integrate TM but notes that implementation has been uneven, and most Pacific Island TM is continues to be practiced largely outside the formal health system, reflecting historical rejection by biomedical services and limited institutional mechanisms for collaboration. Some states to codify traditional healing in law (e.g., Hawai’i, Marshall Islands, Samoa) [73,74]. In Hawai’i, the Native Hawaiian Health Care Act established five Native Hawaiian Healthcare Systems. Commercial and mutual benefit insurers are to cover traditional Native Hawaiian healing and cultural practices Across the broader Pacific; however, direct reimbursement pathways for Oceanic traditional medicine remain limited [75,76].	1.4–1.8 M potential users, some published estimates are 60–80% of the population engaging with TM [76].	Various sources report strong diasporic maintenance of traditional practices [77,78,79]. Kava is one example of Oceanic medicines/practices extending across Pacific, diasporic and wider non-Pacific communities [33,80].	[33,73,74,75,76,77,78,79,80]

**Table 2:** **Examples of sex-based variation in phytomedicine safety or** **efficacy**.

Formulation	TIMS	Target Indication	Proposed Mechanism	Sex-Based Differences in Safety or Efficacy Identified
Sex-based differences in a Kampo gut health medicine				
Daikenchuto (TU-100)	Kampo	Intestinal motility	TU-100 changes gut microbiota composition in mice and increases bioavailability of bacterial ginsenoside metabolites	Observations: Differences in the level of phylum, genus and OTU were established in fecal microbiota between sexes in mice treated with TU-100. *Turicibacter* OTUs increased in females, decreased in males [139]. Implications: *Turicibacter* are higher in tumor-bearing mice, and *Turicibacter* correlates to hepatocellular carcinoma susceptibility; Sexually dimorphic effects may mean high efficacy of TU-100 in female animals could elevate long-term increased cancer risk [140].
Sex-based difference in a Kampo neuroprotective medication				
Geissoschizine methyl	Kampo	Nausea, insomnia, epilepsy, behavioral symptoms dementia	GM has neuroprotective effects against glutamate-induced cell death by reducing ROS generation in the mitochondria and is antiepileptic through inhibiting voltage gated ion channels [141]	Observations: GM shows differences in plasma pharmacokinetics and hepatic metabolism [142] between sexes, with maximal circulating concentrations of GM higher in rat females than males. Implications: High plasma GM is associated with propensity to cross the blood–brain barrier to achieve more immediate neuroprotection [143], and so sex-based differences in dosing and efficacy are therefore likely with GM.
Sex-based difference in efficacy side effect profiles of a traditional Pacific anti-anxiolytic				
Kava *(Piper methysticum)*	Traditional Pacific Medicine	Anxiety	GABA-R agonism by kavalactones	Observations: Sarris et al. [144] investigated the gender differences in the side effects of kava. Women developed higher plasma levels of kavalactones than men and experienced higher frequency and intensity of reported side effects (headaches, nausea, and dizziness) than men. Implications: Potentially differential risk profiles for women and men using Kava as a therapy.
Sex and hormones modulate physiological responses to medicinal Cannabis				
*Cannabis sativa*		Pain, glaucoma, epilepsy, and numerous other indications	Cannabinoid receptor agonism, Transient Receptor Potential ion channel agonism	Observation: Kluger et al. (2015) investigated the sex-dependent differences in the effectiveness of medical cannabis for Parkinson’s disease (PD). Men reported greater improvement in motor symptoms, women reported greater improvement in non-motor symptoms, and side effect profiles and intensity also varied by sex [145]. Implications: Sex-based differences in efficacy and side effects may correlate with differences in hormones between sexes (in animal models, cannabis suppresses gonadal steroids, growth hormones, prolactin and thyroid hormones but activates the hypothalamic–pituitary–adrenal axis [146]).
Sex-specific effects of a S. American phytomedicine for gastric ulcer healing				
*Eugenia punicifolia* (HEEP)	Traditional South American Medicine	Gastric ulcer healing and gastroprotective activities	HEEP mediates prostaglandin E_2_ in male rats and decreases Caspase-8 and Bcl-2 in intact females vs. ovariectomized females and males	Observation: Périco et al. [147] showed that treatment with HEEP on intact female rats reduced ulcerative lesions significantly more (85.22%) than in ovariectomized females (65.47%) and male rats (52.44%). Implications: The sex-specific effects seen here are thought to be modulated by female sex hormones.
Gender-specific safety profile of a traditional fertility treatment				
*Eriosema laurentii* (Leguminosae)	Traditional African Medicine	Treatment for infertility and menopause	Leguminosae has estrogenic properties, and aryl hydrocarbon receptor agonistic behavior [148]	Observation: Ateba et al. [149] did not observe acute toxicity of Leguminosae in Wistar rats, but an immunosuppressive effect was noted in male rats that was not seen in female rats during a 28-day oral toxicity study. Implications: Longer-term exposure to this Traditional African Medicine may have sex-specific impacts on the immune system.
Sex-specific toxicity of traditional epilepsy, pain, and insomnia treatment				
*Dalbergia saxatilis*	Traditional African Medicine	Epilepsy, pain and insomnia treatment	Alpha 2-adrenergic receptors [150]	Observation: Dose-related hair loss was reported [151] in male rats but not in female rats, while female rats showed non-reversible reduced eosinophil and monocyte counts. Male rats experienced reduced sperm count after treatment. Implications: Exposure to this Traditional African Medicine may have sex-specific impacts on white blood cell counts and immunocompetence in females.
Sex-specific effects on longevity regulation				
*Prunella vulgaris*	Traditional Chinese Medicine	Extending lifespan	Nuclear factor E2 (Nrf2), Hsp70	Observation: Female *Drosophila* showed [152] extended lifespans by 10.42% and improved endurance under heat stress by 18.46%, with no significant changes seen in male drosophila. Implications: Further study required in mammalian and human systems to identify potential sex-specific efficacy.
Sex-specific effects on dyslipidemia treatments				
Berberine	Traditional Chinese and Ayurvedic Medicine	Dyslipidemia (high cholesterol)	Reduced proprotein convertase subtilisin/kexin type 9 (PCSK9) mRNA and plasma protein [153]	Observation: Berberine showed significant reduction in males and females in total cholesterol, LDL cholesterol, triglycerides and Apolipoprotein B [154]. Women showed a significant increase in HDL cholesterol while men did not show the same increase. Implications: Potential sex-specific differential of dyslipidemia therapy.

**Table 3:** **SAGER guidelines and their limitations in the phytomedicine** **field**.

SAGER Guidelines	Potential Limitations and Considerations in TIMS settings	
	Limitations	Considerations
1. Design studies that are sufficiently powered to answer research questions for both males and females if the health condition being studied occurs in all sexes and genders	Inclusivity: Incorrectly asserts that males and females comprise ‘all sexes and genders’	Problematic for resource-limited research communities associated with research in many settings for TIMSs; Potentially unachievable in cultures where women and non-binary rights are oppressed; Dismisses observational and case study evidence types that are common practices in TIMS settings; Places exclusionary barriers to participation on traditional practitioners who operate outside the academic mainstream; Variability in practice (individualized medicine) is a cornerstone of many TIMSs and may impact capacity to report standardized treatments or generalized effects; Imposes Western universalist views of gender equity on TIMS settings where cultural sensitivities, norms and understandings of sex and gender may be context-specific.
2. Provide sex- and/or gender-specific data where relevant in all clinical, basic science and epidemiological studies	Definitional Variance: In many traditional cultures, definitions and roles of sex and gender are not strictly binary and may not align with Western classifications. Collecting and categorizing data according to a binary or even a biopsychosocial model of sex and gender could misrepresent the reality of these communities. In general, operant definitions of sex and gender should be defined in all settings and studies, especially where there is a continuum of ways of being (e.g., non-binary). Relevance of Sex and Gender: Not all traditional medicine practices differentiate treatments based on sex or gender. Thus, the forced application of these categories might not yield meaningful data or could oversimplify complex traditional practices that have their own valid systems of patient differentiation.	
3. Analyze the influence (or association) of sex or gender on the results of the study, or indicate and discuss why such analyses were not performed		
4. If sex or gender analyses were performed post hoc, indicate that these analyses should be interpreted cautiously	Privileges settings with resources to initiate *de novo* studies and disenfranchises secondary data analyses	

## Data Availability

The original contributions presented in this study are included in this article. Further inquiries can be directed to the corresponding author.

## Abbreviations

SBV sex as a biological variable; TIMS Traditional and Integrative Medical Systems; RCT randomized controlled trial; FDA U.S. Food and Drug Administration; NIH U.S. National Institutes of Health; EMA European Medicines Agency; CIHR Canadian Institutes of Health Research; GAO U.S. Government Accountability Office; ADR adverse drug reaction; WHO World Health Organization; NCCIH National Center for Complementary and Integrative Health (U.S.); NSDUH National Survey on Drug Use and Health; SOGI sexual orientation and gender identity; SGM sexual and gender minority; LGBTQIA+ lesbian, gay, bisexual, transgender, queer/questioning, intersex, asexual/aromantic/agender, plus others; AIM-AHEAD Artificial Intelligence/Machine Learning Consortium to Advance Health Equity and Researcher Diversity; SAGER Sex and Gender Equity in Research; TCM Traditional Chinese Medicine; TKM Traditional Korean Medicine; PCSK9 proprotein convertase subtilisin/kexin type 9; LDL low-density lipoprotein; HDL high-density lipoprotein; CYP3A4 cytochrome P450 3A4; CYP1A2 cytochrome P450 1A2; ROS reactive oxygen species; OTU operational taxonomic unit; Nrf2 nuclear factor erythroid 2-related factor 2; Hsp70 heat shock protein 70; GABA-R gamma-aminobutyric acid receptor; TRP transient receptor potential (ion channel); HEEP hydroalcoholic extract of *Eugenia punicifolia*; LAR Largehead Atractylodes Rhizome; DEIA diversity, equity, inclusion, and accessibility; CAM complementary and alternative medicine; EU European Union; US United States.

## Glossary

Sex: Biological classification of living organisms based on chromosomal composition, gonadal structure, and hormone profile. In cell-based research, “sex” refers to the chromosomal complement (e.g., XX or XY) of the cell line from which the sample originated. Gender: A sociocultural construct referring to roles, behaviors, and identities associated with being male, female, or another gender identity. Relevant in clinical and epidemiological studies where social context influences exposure, treatment access, and health outcomes. Cell Line Sex: The biological sex of the original donor from which a cell line was derived, typically determined by chromosomal analysis or source documentation. Reporting this variable allows identification of sex-specific cellular responses to experimental conditions. Donor Sex (Clinical Samples): The biological sex of the human participant or tissue donor, as recorded in medical or registry data. Important for identifying sex-differentiated effects in pharmacological and toxicological studies. Gender Identity (Clinical Registers): The self-reported or recorded gender of a participant, which may not align with biological sex.
